# Folding of Matrix Metalloproteinase-2 Prevents Endogenous Generation of MHC Class-I Restricted Epitope

**DOI:** 10.1371/journal.pone.0011894

**Published:** 2010-07-30

**Authors:** Virginie Renaud, Emmanuelle Godefroy, Pierre Larrieu, Fabrice Fleury, Francine Jotereau, Yannick Guilloux

**Affiliations:** 1 Institut National de la Santé et de la Recherche Médicale, UMR 892, Nantes, France; 2 Cancer Institute, New York University School of Medicine, New York, New York, United States of America; 3 Faculté des Sciences et Techniques de Nantes, Nantes, France; 4 Centre nationale de la Recherche Scientifique Unité de Biotechnologie, Biocatalyse et Biorégulation, Nantes, France; Karolinska Institutet, Sweden

## Abstract

**Background:**

We previously demonstrated that the matrix metalloproteinase-2 (MMP-2) contained an antigenic peptide recognized by a CD8 T cell clone in the HLA-A*0201 context. The presentation of this peptide on class I molecules by human melanoma cells required a cross-presentation mechanism. Surprisingly, the classical endogenous processing pathway did not process this MMP-2 epitope.

**Methodology/Principal Findings:**

By PCR directed mutagenesis we showed that disruption of a single disulfide bond induced MMP-2 epitope presentation. By Pulse-Chase experiment, we demonstrated that disulfide bonds stabilized MMP-2 and impeded its degradation. Finally, using drugs, we documented that mutated MMP-2 epitope presentation used the proteasome and retrotranslocation complex.

**Conclusions/Significance:**

These data appear crucial to us since they established the existence of a new inhibitory mechanism for the generation of a T cell epitope. In spite of MMP-2 classified as a self-antigen, the fact that cross-presentation is the only way to present this MMP-2 epitope underlines the importance to target this type of antigen in immunotherapy protocols.

## Introduction

Over the last 20 years, several human tumor antigens recognized by autologous cytolytic T lymphocytes (CTL) have been characterized. Four classes of tumor antigens can be distinguished: cancer testis antigens [1], [2] differentiation antigens [3]–[5] mutated antigens [6] and antigens aberrantly expressed in tumors [7], [8]. To our knowledge, little is known about the immunogenicity of these antigens. One of the objectives of our work is to define tumor antigens involved in tumor regression after cancer treatments [9], [10].

We recently described the recognition, on about half HLA-A2 melanoma cell lines, of a new HLA-A2-restricted tumor antigen derived from Matrix Metalloproteinase 2 (MMP-2) by a CTL clone (M134.12) derived from Tumor Infiltrating Lymphocytes (TILs) of a melanoma patient [9]. We showed that recognition of melanoma cells by this CTL clone required MMP-2 secretion and cross-presentation of the HLA-A2-restricted MMP-2_560–568_ epitope following αvβ3-dependent endocytosis of secreted MMP-2 [9]. Surprisingly, the MMP-2 epitope, located in the C-terminal extremity of the protein, was poorly or not processed by the classical endogenous pathway, since melanoma cells, which secrete MMP-2 but lack αvβ3 integrin expression, and cells that synthesized MMP-2 but do not secrete it, failed to be recognized by the specific CTL clone [9].

MMP-2 belongs to the gelatin-binding MMPs structural group. This enzyme contains an amino-terminal signal sequence (Pre) that directs it to the secretory pathway via the endoplasmic reticulum (ER) and a propeptide (Pro) that maintains it as inactive zymogen [11]. MMP-2 comprises three contiguous fibronectin type II-like domains that are inserted within its catalytic domain, one hemopexin domain, and eight disulfide bridges (*NCBI number P08253*) [12]. MMP-2 secreted by normal and tumor cells has been shown to play a key role in angiogenesis, tumor cell invasion, and metastasis, by promoting degradation of Extracellular Matrix (ECM) and cleavage of cytokines, growth factors, hormones, and cell receptors [13]–[16]. Because of its role in melanoma progression, targeting the MMP-2 antigen by immunotherapy might be particularly effective by limiting the potential selection of antigen loss variants. Since many normal cells express MMP-2, it is important to understand the mechanisms that may prevent its endogenous presentation. To this end, using the MMP-2 CTL clone we addressed which alterations of the MMP-2 cDNA would lead to endogenous presentation of the MMP-2_560–568_ epitope upon transfection.

In this report, we provide evidence that disulfide bonds contained in the MMP-2 protein prevent endogenous presentation of the appropriate HLA-A2-restricted epitope. Furthermore, we demonstrate that wild type MMP-2 is less sensitive to degradation than mutated forms obtained by disulfide bond deletion. Altogether these results identify a novel mechanism underlying selective tumor cell recognition through cross-presentation of a post-translationally modified ubiquitous protein.

## Results

### Deletion of the first 96 amino acids of MMP-2 restores classical endogenous presentation of the MMP-2_560–568_ epitope

**Figure 1:**
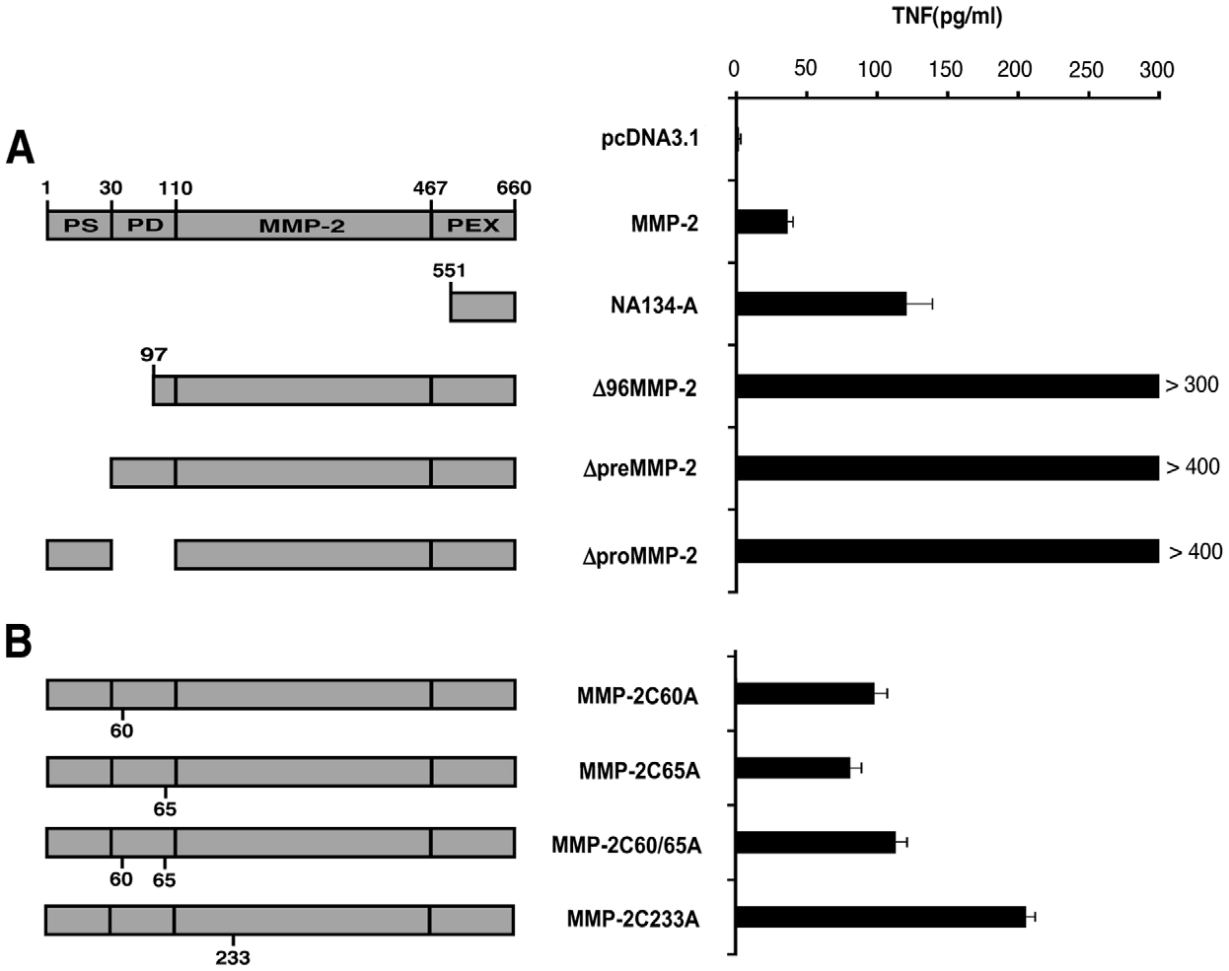
Various deletions and mutations of MMP-2 cDNA allow MMP-2_560–568_ epitope generation by the endogenous pathway in HLA-A*0201 transfected COS-7 cells. COS-7 cells were cotransfected with HLA-A*0201 plasmid and with (A) plasmids coding for deleted MMP-2 or (B) plasmids coding for mutated MMP-2. 48 h later, M134.12 CTL clones were added to transfected COS-7 cells (E/T ratio 1∶3) and the TNF response was tested after 6 h on Wehi-13 cells. Standard deviations were obtained from duplicates. cDNA NA134-A corresponding to the C-terminal part of MMP-2 contains MMP-2_560-568_ epitope and was used as positive control. Transfection efficiency was controlled with GFP transfected COS-7 cells. PS corresponding to the signal sequence (pre), PD corresponding to the prodomaine (pro) and PEX corresponding to the hemopexine domaine of the MMP-2. Data are representative of at least two independent experiments. Error bars indicate standard deviations of duplicates.

We previously described the recognition by a TIL-derived clone of a MMP-2 derived HLA-A2-restricted peptide cross-presented by αvβ3+ tumor cells. This clone, however failed to react against αvβ3-negative HLA-A2+ cells expressing MMP-2, thus indicating inefficient presentation of the MMP-2_560–568_ epitope by the endogenous pathway. To address the underlying mechanisms of these observations, we made progressive deletions of the MMP-2 cDNA from the 5′ to the 3′ end by PCR. COS-7 cells co-transfected with plasmids coding for truncated variants of MMP-2 and HLA-A*0201 were then tested for their ability to stimulate TNFα release from the relevant CTL clone (M134.12). The minimally deleted construct thus obtained, corresponding to the cDNA with a 1 to 96 aa deletion (Δ96MMP-2) restored expression of the MMP-2 epitope (fig. 1A). This suggests that the region 1–96 of MMP-2 can impede endogenous presentation of the MMP-2 epitope localized in the C-terminal extremity of the protein. Because sequence 1–96 of MMP-2 includes the secretion peptide and part of the propeptide, we hypothesized that either the compartmentalization of MMP-2 in the secretory pathway affects routing to the proteasome [17] or that the propeptide contains an inhibitory sequence blocking proteasomal degradation [18].

### Deletion of the signal sequence or the propeptide restores classical endogenous presentation

To address these two hypotheses, we constructed by PCR two deleted forms of MMP-2 cDNA. One form harbored a signal peptide deletion (Δ Pre) and the other, a deletion of the region encoding the propeptide (Δ Pro). Both cDNAs (Δ Pre and Δ Pro) led to effective MMP-2 epitope presentation by HLA-A*0201-transfected COS-7 cells and subsequent CTL clone activation (Fig. 1A). A potentially relevant shared feature of both MMP-2 products (Δ Pre and Δ Pro) is the absence of disulphide bonds. Propeptide deletion (Δ Pro) eliminates a disulfide bridge (Cys^60^ to Cys^65^) that is unique to proMMP-2 [12]. Moreover signal peptide deletion (Δ Pre) should prevent MMP-2 translocation across the endoplasmic reticulum (ER) and subsequent disulfide bond formation. Therefore the efficient endogenous presentation of MMP-2_560–568_ epitope from Δ Pre and Δ Pro constructs could be due to misfolding of the truncated MMP-2 protein, resulting from the absence of at least one disulfide bond.

### Disulfide bond formation inhibits MMP-2_560–568_ endogenous presentation

To address the role of disulfide bond formation in modulation of MMP-2 endogenous presentation more directly, we generated mutants in which the disulfide bond contained in the prodomain was disrupted. To this end we substituted cystein 60 and/or cystein 65 by an alanine. All mutated MMP-2 constructs (MMP-2 C60A or MMP-2 C65A or MMP-2 C60A/C65A) allowed efficient endogenous expression of the MMP-2 antigen when cotransfected with HLA-A2.1 plasmids into COS-7 cells (Fig. 1B). To determine whether this disulfide bond (Cys^60^ to Cys^65^) had a specific role in preventing MMP-2 endogenous presentation, we mutated the cystein codon of MMP-2 protein involved in the formation of another disulfide bond (Cys^233^ to Cys^247^) located in the first fibronectin domain of MMP-2 (MMP-2 C233A). As shown in Fig. 1B, this mutated plasmid also induced MMP-2 epitope presentation by COS cells. Therefore endogenous presentation of the MMP-2 antigen can be induced by mutations impeding the formation of one disulfide bond within MMP-2 propeptide or fibronectin domain.

### Elimination of one disulfide bond restores MMP-2 endogenous presentation in human tumor cell lines

**Figure 2:**
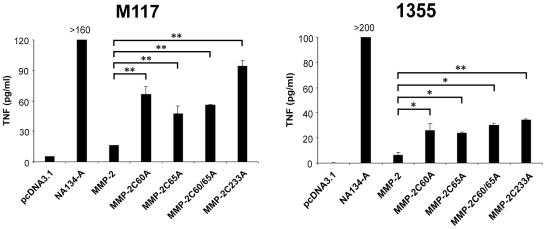
Disulfide bond deletion permit MMP-2_560–568_ epitope generation by the endogenous pathway in HLA-A*0201+/αvβ3- human tumor cells. Melanoma cell line M117 and non small cell lung carcinoma line 1355 were transfected with plasmids coding for cystein deleted MMP-2. 48 h later, M134.12 CTL clones were added to tumor cells (E/T ratio 1∶3) and the TNF response was tested after 6 h on wehi-13 cells. Standard deviations were obtained from duplicates. cDNA NA134-A corresponding to the C-terminal part of MMP-2, contains MMP-2_560–568_ epitope and was used as positive control. Transfection efficiency was controlled with GFP transfected tumor cells. Data are representative of at least two independent experiments. Error bars indicate standard deviations of duplicates. p<0.005 was considered significant.

In order to determine whether the endogenous presentation of MMP-2 by tumors cells is similarly affected by the removal of disulfide bonds, we used HLA-A2 tumor cell lines that fail to cross-present MMP-2 due to their lack of αvβ3 integrin [9]. We previously showed that the expression of the αvβ3 integrin is necessary for exogenous presentation of MMP-2 antigen. Transfection of plasmids encoding the four mutated forms of MMP-2 (MMP-2C60A, MMP-2C65A, MMP-2C60/65A and MMP-2C233A) into the M117 melanoma cell line or the non-small cell lung carcinoma (NSCLC) 1355 cell line, induced activation of the MMP-2- specific CTL clone (Fig. 2). Therefore, as in COS-7 cells, endogenous presentation of the MMP-2_560–568_ epitope in human cells can be induced by mutational deletions impeding formation of a single disulphide bond within this protein.

### Disulfide bond mutation does not impede MMP-2 secretion

**Figure 3:**
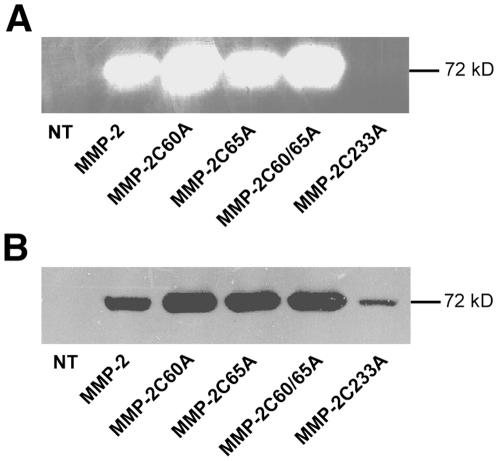
Cystein deletion does not impede MMP-2 release. COS-7 cells transfected with indicated plasmids were cultured for 48 h without FCS. Supernatants were collected and equal amounts of protein were loaded on SDS-PAGE. MMP-2 release was analyzed by (A) gelatin zymographie and (B) western blotting. *NT*: untransfected COS-7 cells. Data are representative of at least two independent experiments.

To determine whether disulfide bond deletion could affect MMP-2 protein secretion, we transfected MMP-2 cDNA with mutated Cys codon into COS-7 cells. 48 h later, culture supernatants were harvested and MMP-2 secretion was assessed by detecting the enzymatic activity of MMP-2 by zymography (Fig. 3A). All mutated proteins, with the exception of MMP-2 C233A, showed MMP-2 activity. Moreover western blotting indicated that all mutated variants led to protein secretion (Fig. 3B) even though MMP-2 C233A had no MMP-2 activity (Fig. 3A). Therefore protein secretion does not impede endogenous presentation of the MMP-2_560–568_ epitope. However, the fact that MMP-2 C233A, which was secreted at a lower level (Fig. 3B) induced higher clone activation (Fig. 1B), suggests that protein secretion may nonetheless limit endogenous antigen presentation.

### Protein turn over is higher in Mutated MMP-2 mutants versus WT MMP-2

**Figure 4:**
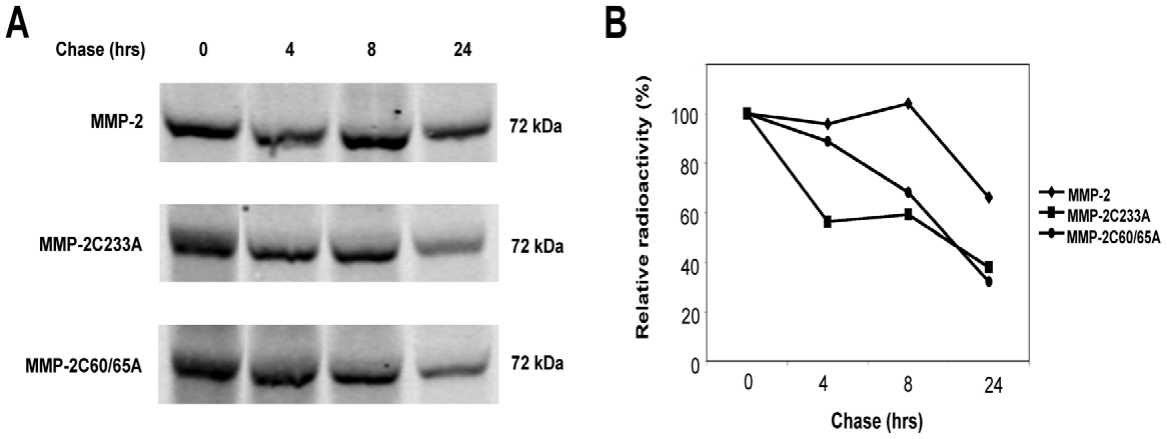
Mutated MMP-2 proteins are more rapidly degraded than the wild-type enzyme. COS-7 cells transfected with indicated plasmids were pulse-labeled with [^35^S] methionine/cystein for 15 min and chased for 0–24 h. MMP-2 immunoprecipitates were separated by SDS-PAGE and analyzed by autoradiography (A). Data were plotted to indicate the residual protein remaining where the amount of this protein at 0 h time point was calculated to represent 100% of total MMP-2 in each case (B). Data are representative of at least two independent experiments.

The fact that disruption of a single disulfide bond within MMP-2 was sufficient to induce endogenous presentation of the MMP-2 epitope, suggests that decreased conformational stability of MMP-2 due to impaired disulfide bond formation lead to enhanced endogenous degradation and subsequent MMP-2_560–568_ epitope presentation. To test this hypothesis, we sought to determine whether disulfide bond integrity affects MMP-2 stability. Cells were metabolically labeled with [^35^S] methionine/cystein for 15 mn, followed by a 24 h chase. As shown in Fig. 4A and 4B, wild type MMP-2 was stabler than mutated MMP-2. Indeed, after 8 h, approximately 40% of mutated forms were degraded (31.8% for MMP-2C60/65A and 40.8% for MMP-2C233A) whereas wild type MMP-2 was still intact. After 24 h, more than 60% of each mutated forms were degraded (67.9% for MMP-2C60/65A and 62.3% for MMP-2C233A) whereas the wild type protein was degraded about twice as less (33.9%). These results suggest that mutated MMP-2 forms are more sensitive to degradation than the wild type protein is.

### MMP-2 epitope presentation from mutated MMP-2 constructs is mediated by the proteasome and depends on a retro-translocation channel

**Figure 5:**
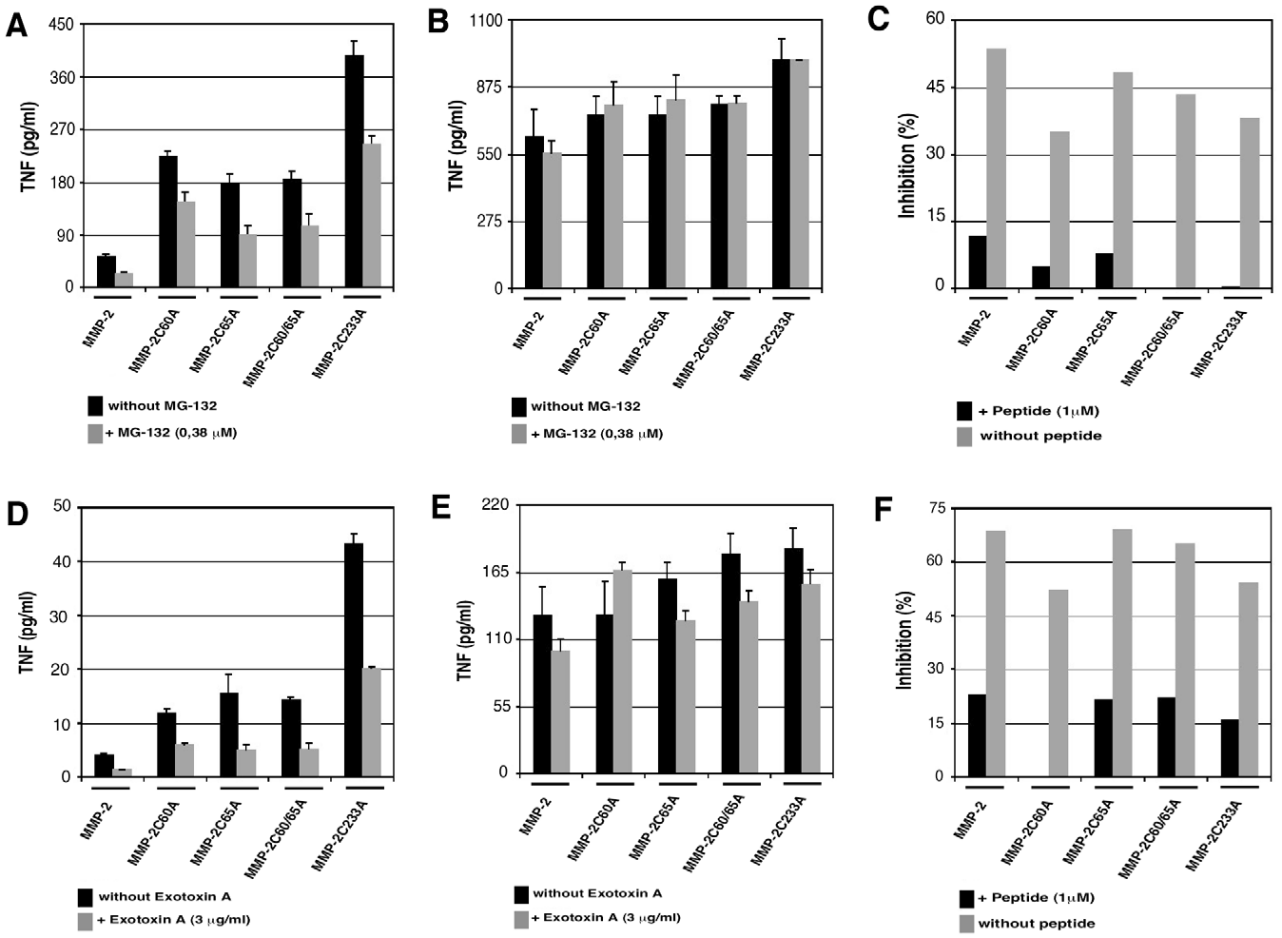
MMP-2 endogenous presentation require proteasome and sec61. *A and D*: COS-7 cells were cotransfected with plasmids coding for WT MMP-2 or mutated MMP-2 and for HLA-A*0201. 24 h later, COS-7 cells were treated with proteasome inhibitor MG-132 (A) or sec61 inhibitor Exotoxin-A (D). After overnight incubation, cells were washed, counted and cocultured with M134.12 CTL clones (E/T ratio 1∶3). The TNF response was tested 6 h later on wehi-13 cells. *B and E*: cells treated as in A and D, were pulsed 1 h with peptide before coculture with M134.12. Peptide, corresponding to the MMP-2_560–568_ epitope, was used as control of HLA-A*0201 surface expression. *C and F*: Inhibition percentages are expressed as the percent TNF response of cells treated with drugs versus the percent TNF response of cells untreated, where TNF response of cells untreated was normalized to 100%. Transfection efficiency was controlled with GFP transfected COS-7 cells. Data are representative of two independent experiments. Error bars indicate standard deviations of triplicates.

Misfolded secretory proteins undergo retrograde translocation from the ER and are then hydrolyzed by the ER-associated degradation pathway (ERAD) [19], [20]. Two important components of ERAD are the ubiquitin-proteasome system and the retro-translocation channels allowing the transport of misfolded proteins from the ER to the cytosol [21]–[23]. To ascertain whether MMP-2 epitope presentation from the MMP-2 mutants was dependent on these ERAD components, MMP-2 transfected COS cells were treated with the proteasome inhibitors MG-132 or by the retrotranslocation channels inhibitor: *Pseudomonas aeruginosa* Exotoxin-A [24], [25]. Both inhibitors inhibited MMP-2 epitope presentation from MMP-2 constructs, however they had no effect on the presentation of added synthetic 9-mer MMP-2 peptide (Figure 5A and B). These results indicate that the degradation rate of mutated MMP-2 regulated by the ERAD machinery directly affects MMP-2 immunogenicity.

## Discussion

In this current work, we investigated the mechanisms underlying the lack of MMP-2 epitope (560–568) presentation via the classical endogenous pathway by MMP-2 cDNA construct transfection into COS-7 cells and into tumor cells. In this pathway, antigenic peptides derived from endogenous proteins intersect the MHC class I biosynthetic pathway in the ER, where MHC class I heavy chain and β_2_-microglobulin are synthesized. These peptides are transported from the cytosol to the ER via the transporter associated with antigen processing (TAP) complex and loaded on nascent MHC class I molecule and β_2_-microglobulin complexes [26], [27]. Surprisingly we previously reported that MMP-2 failed to be presented by this classical endogenous pathway [9].

In 1–96, pre or pro deleted MMP-2, we eliminated a quite high quantity of amino acids compared to mutated MMP-2, where only one cysteine was replaced by an alanine. This probably induces a higher deleted proteins misfolding compared to mutated proteins. This can explain why deleted proteins have significantly better endogenous presentation of MMP-2 antigen than cysteine mutants. However, in this paper we showed that elimination of only one disulfide bond in the MMP-2 protein is sufficient to induce endogenous presentation of the MMP-2_560–568_ epitope. Further more, we showed that the wild type MMP-2 protein is less degraded than the mutated forms. We analyzed these proteins by circular dichroism (CD). The results showed that the spectra of MMP-2 mutants are found to be practically identical to the spectrum of wild-type MMP-2 (data not shown). So, the CD analysis confirms the protein folding and shows that the mutations do not induced significant changes in the secondary structure of mutated MMP-2. However, this absence of changes in secondary structures does not exclude other conformational changes in the whole MMP-2 protein. Indeed, based on MMP-2 crystal structure (Morgunova et al., 1999) we learnt that disulfide bond C60–C65 is located in the loop between the helix H1 and H2 of the prodomain. This prodomain maintained MMP-2 as a zymogen and this loop is cleaved by MMP-14 after MMP-2 secretion given active form. Moreover disulfide bond C233–C247 is located in the first Fibronectin type II domain and this domain is necessary for substrate binding. So these two disulfide bonds seem to be located in important regions for wild type MMP-2. These observations and our experiments probably mean that, for wild type MMP-2, quality control of these two regions is essential, so that none, or few, misfolded enzymes are naturally produced and consequently no, or few, MMP-2 epitope presentation occur. Altogether these results suggest that MMP-2 folding has an inhibitory role on the endogenous degradation of MMP-2 by the proteasome and its subsequent immunogenicity.

Disulfide bond formation in the endoplasmic reticulum of eukaryotic cells is catalyzed by the protein disulfide isomerase (PDI) and other members of the thioredoxin family [28]–[30]. Disulfide bond formation takes part in protein folding, stability, and activity [31]–[33]. Other studies provided molecular evidence that intact disulfide bonds are critical for proteasome resistance. Elimination of disulfide bonds in murine Proinsulin 2, in bovine viral diarrhea virus E2 glycoproteins as well as in Human ABCG2 render these proteins sensitive to proteasomal degradation. Nonetheless to our knowledge no proteasome derived peptides from these proteins (murine Proinsulin 2, BVDV E2 glycoproteins and human ABCG2) have been identified as targets of CTL [34]–[36]. Even if we already know that disulfide bridges impede proteasome degradation, it is the first time a disulfide bridge is shown to impeding CD8 T cell epitope generation.

In a similar way, the structural complexity of a protein and especially the presence of disulfide bonds, seems to be critical for MHC class II presentation. Indeed disulfide bond reduction of proteins facilitates processing and presentation to antigen-specific CD4 T cells [37]–[40]. To our knowledge little is known about the impact of disulfide bond formation on endogenous MHC class I presentation. The classical class I antigen processing pathway is considered independent from 3D structure constraints since the antigen pools from which MHC class I epitope are derived, mainly come from unfolded protein or DRiPs [41]–[43]. Our previous observation that a fraction of HLA-A*0201 tumor cell lines lacking αvβ3 integrin expression and secreting MMP-2 protein was not recognized by the specific T cell clone, led us to suggest that the amount of unfolded form or DRiP containing the epitope are low in these cells. However since our functional read-out involved a T cell clone specific for an epitope localized in the C-terminal extremity of MMP-2, we cannot formally rule out formation of DRiPs or misfolded proteins from the N-terminal extremity of MMP-2.

By preventing the formation of at least one disulfide bond, we demonstrated that the redox status of MMP-2 significantly increased its degradation and resulted in processing of the specific T cell epitope by the endogenous pathway. Furthermore it did not prevent MMP-2 secretion. Based on these findings, we propose that disruption of one disulfide bond is detected by the quality control mechanism present in the ER [22], [44]. This mutation would entail the reimportation of mutated MMP-2 from the secretory pathway to the cytosol, where the protein is processed by the ER-associated degradation (ERAD) machinery [19], [22], [45]. To our knowledge these data would indicate that protein folding may affects endogenous class I restricted T cell epitope generation.

**Figure 6:**
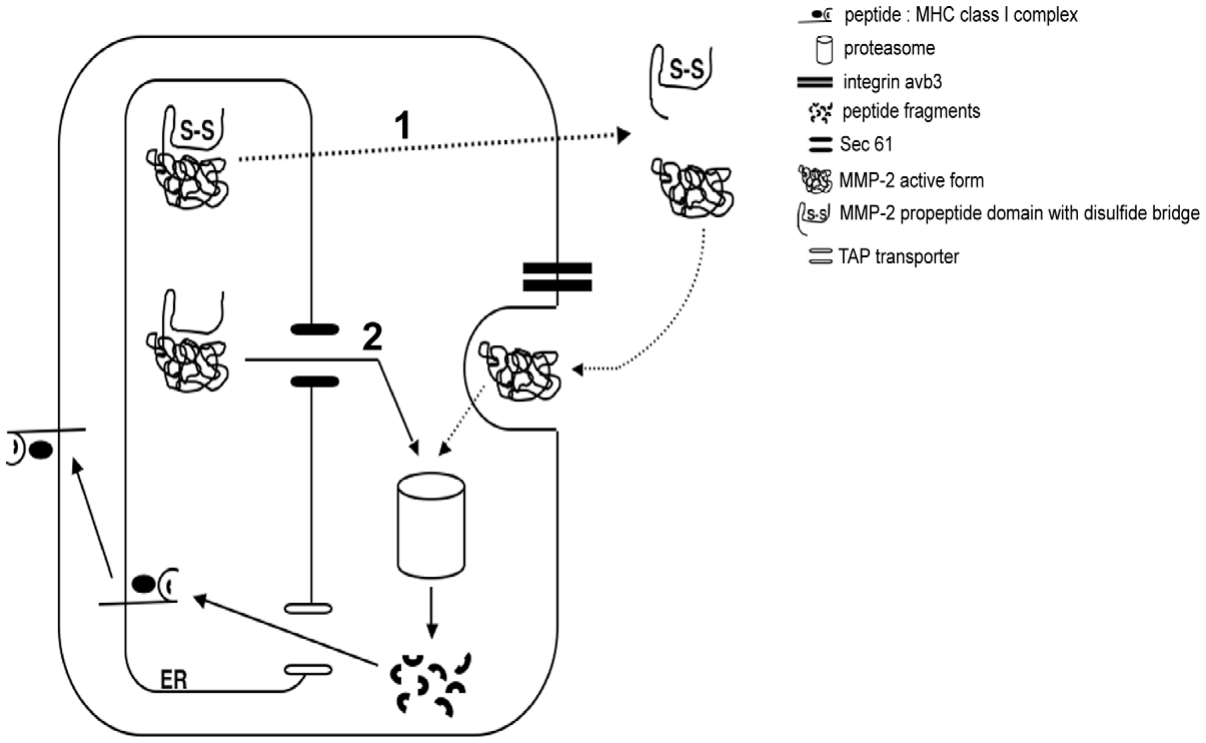
Hypothetical models for MMP-2_560–568_ epitope generation by cross-presentation and by endogenous pathway. Cross-presentation (1): Newly synthesized wild-type MMP-2 acquire disulfide bonds in the endoplasmic reticulum (ER) before joining the secretory pathway. In the extracellular environment, physiologic activation of the pro-MMP-2 induce the cleavage of the propeptide domain which contains a disulfide bridge (C60-C65: unique to the MMP-2). MMP-2 active form then interact with the integrine αvβ3 and is internalized in clathrin-coated vesicle. Finally MMP-2 is transported to the cytosol, in an unknown mechanism, and degraded by the proteasome. Peptides generated can reach the endogenous pathway (peptides are transported in the ER through TAP, bind to HLA-A*0201 and transported to the cell surface). Endogenous presentation (2): Mutated MMP-2 lacking a disulfide bond can't join the secretory pathway and is retrotranslocated via Sec61. In the cytosol, mutated MMP-2 is degraded by the proteasome and resulting peptides are loaded on MHC class I molecules.

According to this hypothesis, we propose in Fig. 6 a model explaining why the MMP-2 epitope is normally presented only through the cross-presentation pathway. In such a model, cells secrete MMP-2 as an inactive zymogen (Pro-MMP-2) containing 8 disulphide bonds [11], [12]. Owing to its highly stable conformation, Pro-MMP-2 shows very limited degradation through the ERAD pathway. Pro-MMP-2 is activated at the cell surface through a multistep pathway that involves MMP-14 (MT1-MMP) and the tissue inhibitor of metalloproteinases 2 (TIMP-2) [46]. Pro-MMP-2 might also be activated by MMP-15 through a TIMP-2-independent mechanism [47]. Pro-MMP-2 activation is associated with proteolytic removal of the propeptide prodomain including Cystein 60–65 disulfide bond [48]. In this regard, this activated form could be detected by zymography in supernatants of all melanoma cell lines that cross-presented the MMP-2 epitope 560–568 (Godefroy personal communication). Activated MMP-2 containing only 7 disulfide bonds is then internalized in clathrin-coated vesicles and subsequently by an unknown mechanism reachs the cytosol for proteasome degradation. During protein synthesis, MMP-2 acquired 8 disulfides bond in the ER, probably impeding its retrotranslocation and its degradation by proteasome and subsequently MMP-2_560–568_. epitope presentation by the endogenous MHC class I pathway.

MMP-2 is a self-antigen secreted by many cells and involved, through the degradation of the extracellular matrix and basal membranes, in multiple physiological processes and in tumor progression. Nevertheless, the MMP-2 epitope was found to be cross-presented exclusively by melanoma cells and not presented by the endogenous pathway [9]. All these characteristics point to MMP-2 as the target of interest in melanoma immunotherapy protocols.

## Materials and Methods

### Cell lines and culture medium

The CTL clone M134.12, melanoma cell line M117 were established in our laboratory (Godefroy et al 2005). M134.12 was obtained from Tumor Infiltrating Lymphocytes (TIL) of melanoma patient M134 by limiting dilution and selected for it specificity against the antigen MMP-2 in an HLA-A*0201 context. Non-small cell lung cancer cell line (1355) (established by H. Oie) was gift from C. Saï (UMR 892 INSERM/Université de Nantes, France). Mouse fibrosarcoma WEHI 164 clone 13 (established by Rollinghoff and N.L. Warner) and COS-7 cells (established by Y. Gluzman) were gifts from T. Boon (Ludwig Institute for Cancer Research, Brussels, Belgium).

Tumor cell lines and COS-7 cell were maintained in RPMI-1640 and DMEM 1 g glucose/L respectively, supplemented with penicillin-streptomycin (100 U/ml and 10 µg/ml respectively), L-Glutamine (2 mM) and 10% fetal calf serum (all from Sigma-Aldrich). The CTL clone was maintained in RPMI-1640 supplemented with penicillin-streptomycin, L-Glutamine, IL-2 (150 U/ml) and 8% pooled human serum (pHS).

### Ethics Statement

Written consents were obtained from all patients and healthy donors. The local ethics committee “Comité de Protection des Personnes Ouest IV- Nantes” and the “Agence française de sécurité sanitaire des produits de santé” approved all these studies.

### Cloning of cDNA encoding modified MMP-2

NA-134A corresponding to the end of the MMP-2 sequence and containing the epitope was obtained from a cDNA library of melanoma cell line M134. Truncated MMP-2 Δ96MMP-2 was obtained by PCR. ΔpreMMP-2 and ΔproMMP-2 were obtained by deletion by PCR of the nucleotide sequence coding for the signal peptide (Δpre) or the propeptide (Δpro). Point mutations were introduced into the cDNA coding for MMP-2 using directional mutagenesis: site-directed mutagenesis was performed using primers with nucleotide substitutions in order to change cystein in alanine at amino acid 60 and/or 65 and 233.

Each of the cDNA constructs was digested with EcoRI and XbaI, inserted into pcDNA3.1 vector (Invitrogen) and plasmids were electroporated into a bacterial strain (Escherichia coli TOP 10 F').

### Transient transfection of COS-7 cells

We used the DEAE-dextran-chloroquine method as described (Brichard et al. 1993, Coolie et al. 1994).

Briefly: 1,5.10 ^4^/well COS-7 cells were plated in flat-bottom 96 well plates. After 24 h cells were cotransfected with 125 ng of plasmids containing the cDNA coding for the different constructs of MMP-2 and with 125 ng of plasmids containing the cDNA coding for HLA-A*0201. Each transfection condition was performed in duplicate. After 48 h, cells were tested in a CTL stimulation assay. Transfection efficiency was controlled by transfected COS-7 cells with plasmid coding for the GFP and measured by flow cytometry analysis.

### Transfection of COS-7 cells using the Cell Line Nucleofector™ kit V from Amaxa

1.10^6^ COS-7 cells were transfected with 5 µg of plasmid (coding for mutated MMP-2) according to the manufacturer's instructions.

For pulse-chase experiments, 7.10^6^ COS-7 transfected with the same plasmid were pooled in 100 mm dishes and cultured 48 h at 37°C before metabolic labeling.

For zymography and Western blotting assays, 1.10^6^ transfected COS-7 were plated in a 6-well plate. 1 day later, cells were washed and cultured for 48 h in medium without FCS.

### Transient transfection of tumor cell lines using lipofectamine reagent

2.10^4^/well tumor cell lines were plated in flat-bottom 96 wells plates 24 h before transfection. Cells were washed with OPTI-MEM medium (Invitrogen) and were transfected using lipofectamine and Plus reagent (Invitrogen) and 100 ng of plasmid containing the cDNA coding for the mutated MMP-2, in OPTI-MEM. All transfection conditions were performed in duplicate. After 48 h, cells were tested in a CTL stimulation assay. Transfection efficiency was controlled by transfected tumor cells with plasmid coding for the GFP and measured by flow cytometry analysis.

### Treatment of transfected COS-7 cells

COS-7 cells were transiently cotransfected with plasmids coding for mutated MMP-2 and for HLA-A*0201 using DEAE-dextran-chloroquine method. 24 h later, COS-7 cells were treated by addition to the culture medium of 0,38 µM proteasome inhibitor MG-132 (Calbiochem) or 3 µg/ml sec61 inhibitor Exotoxin A (Sigma-Aldrich). After overnight incubation, cells were washed, counted and tested in CTL stimulation assay.

### CTL stimulation assay

Transfected cells were tested for their capability to present a MMP-2 derived HLA-A*0201 and consequently to stimulate the production of Tumor Necrosis Factor (TNF) by the specific CTL M134.12.

1.10^4^ CTL were added to 3.10^4^ stimulator cells (transfected tumoral or COS-7 cells) for 6 h at 37°C. Then, culture supernatants were collected and TNF release by CTL was measured by testing TNF cytotoxicity on WEHI 164 clone 13 cells in a MTT colorimetric assay (Espevik et al, 1986).

### Gelatine substrate gel zymography

Secretion of MMP-2 was evaluated by gelatin zymography, as previously described (Barille et al. 1999).

Supernatants were obtained from transfected COS-7 cells (Amaxa) cultured in DMEM 1 g glucose/L without FCS during 48 h and total proteins concentrations were measured (BC Assay, Interchim). 22,5 µg of total protein were mixed with sample buffer without reducing agent and proteins were separated by SDS-PAGE (7,5% acrylamide gels containing 0,2% gelatin). After electrophoresis, SDS was removed from the gel by an incubation in 2,5% triton X-100 for 1 h at room temperature. Gels were then incubated in a buffer containing 50 mmol/L Tris-HCl, 5 mmol/L CaCl_2_, pH 7,6 for 20 h at 37°C and stained with Coomassie blue R250 for 30 min. Proteolytic activity of MMP-2 was evidenced as a clear band against the blue background of stained gelatin.

### Western blotting

Supernatants were obtained from transfected COS-7 cells (Amaxa) cultured in DMEM 1 g glucose/L without FCS during 48 h and total proteins concentrations were measured (BC Assay, Interchim). 50 µg of total protein were mixed with sample buffer containing reducing agent and proteins were separated by SDS-PAGE (7,5% acrylamide), electrotransferred to polyvinylidene difluoride (PVDF) membrane and blocked 1 h in 1% Western Blocking Reagent (Roche Diagnostic) TBS 1X. Blots were probed overnight at 4°C with a rabbit polyclonal antibody anti-MMP-2 hinge region (Biomol, Tebu-bio SAS, France), washed in TBS 1X, 0,1% Tween-20 and probed with HRP-conjugated anti-rabbit, anti-mouse antibody (Roche). The signal was detected by enhanced chemiluminescence detection (Perbio).

### Pulse-chase experiments, immunoprecipitation and SDS-PAGE

48 h after transfection using the Cell Line Nucleofector™ kit V (Amaxa), 7.10^6^ transfected COS-7 were incubated at 37°C for 1 h30 in cystein and methionine-free DMEM 4,5 g glucose/L (Invitrogen). Cells were then pulsed for 15 min with 350 µCi of [^35^S] methionine/cystein (GE Healthcare), plus Brefeldin A (BFA) 10 µg/ml (Sigma-Aldrich) in 5 ml of DMEM 4,5 g glucose/L without cystein and methionine. Cells were then recovered and redistributed: 1.10^6^ cells in 2 ml of DMEM containing FCS plus BFA, and chased from 0 to 24 h. After chase, cells were pelleted, resuspended in lysis buffer (10 mM Tris-HCl pH 7,6, 150 mM NaCl, 5 mM EDTA, 1 mM PMSF, 2 µg/ml aprotinin, 1% Triton X-100) for 45 min on ice and centrifugated at 10000 g for 30 min at 4°C. Protein concentration in supernatants was measured using bicinchoninic acid (BCA Protein Assay, Pierce, Rockford, IL). 70 µg of lysates were precleared with protein G Agarose (Perbio) and then immunoprecipitated overnight at 4°C with a rabbit polyclonal antibody anti-MMP-2 (H-76, Santa-Cruz, Tebu-bio SAS, France) and protein G agarose. Beads were pelleted, washed and boiled in SDS sample buffer. Proteins in immunoprecipitated samples were separated by SDS-PAGE (7,5% acrylamide) and analyzed by autoradiography.

### Statistical analysis

Statistical analysis was done with InStat 2.01. Data were analyzed using ANOVA test. p<0.005 was considered significant.
